# Musings of a Simpleton

**Published:** 1916-07

**Authors:** 


### MUSINGS OF A SIMPLETON.

BY HIMS ELF.

### *Sob Stuff.*

This is a sad, sad story. No comedy. Charles Chaplin is not here today. It is a tale of a conscientious man and a villain, and here it goes. Those who have tears, prepare to shed them now.

Once upon a time there was a very upright and conscientious dentist. His great aim in life was to give the very best service to his patients. He studied diligently and attended every dental meeting he could run to earth. The more he read and the more he meetinged, the more he became convinced that he was not doing all he should towards his patients. He was doing too much himself, too much that should be done by those better qualified.

So he in his great desire to be honest, made a change. He sent all his extractions to an exodontist (*i. e.*, a high-priced tooth puller) and then his regulating, heavens—he, a general practitioner, had been doing regulating. He sent them to an orthodontist. Then he attended one more meeting and—bang went his root canal work. All bad, he sent it to a rootologist. He came very near buying an X-ray outfit to make sure of his root canal work, but luckily he happened to read of its dangers in inexperienced hands, so off to the radiographist for the patients. His patients, who wore artificial dentures, could never whistle and eat sticky molasses candy with his plates, but, ah-ha, the prosthetist could make them that way, he had heard them say so, so to the expert they went for plates.

This left him his crown and bridge work and he was batting out a poor livelihood making a crown once a week when he happened to hear of specialists in this, so, conscientious he, he had to send them to the best.

One day he was cleaning a set of teeth, scaling the roots and all that, when a guilty thought assailed him. Rushing to his dental literature, he found that it was so. "Patient, get out of my chair. I have no right to be doing this. Your gums bled once. Some day you might have Riggs Disease. Fortunately there is a specialist who does this kind of work. Beat it to him." "And who and what is this in my reception room? A patient with a swollen face, and will I lance that abscess? Not on your gumboil! Who am I that I should do such work? You for the oral surgeon."

And so, after a short time when the last surviving patient came in for an amalgam filling he had to insert it, but, oh, how he trembled for fear that alloy wasn't properly balanced.

Now for the second episode. As the weary patients plodded their lonely ways towards the various specialists, they had to pass the office of the villian of the piece. He was likewise a dentist and he had a sign out with dental surgeon on it.

The specialized patients, with one accord, thought to themselves: "It's a long way to specialdom. I wonder what a dental surgeon is?" And so in the office they went.

Did he send them away? He did not by a great deal. He got busy and filled the teeth, and pulled the teeth, and stuck the abscesses. He also went to dental societies and learned to say apicoectomy without laughing, and then he went back to his office and did it. The name strepto-coccus veridans had no terrors for him. He followed it up with endocarditis and arthritis and showed it all to his patients on his home-made X-ray pictures. He showed gutta-percha points right up to the patients' eyes, and used a system for regulating that was absurd but did the work.

So, with one accord, all the patients said: "See America first. We will see the foreign talent later if we need them." But they never did.

And he engaged assistants, didn't even call them associates, and went merrily on his way until he became rich enough to both own an automobile and play golf. Greater riches hath no man.

Now for the moral. No; there is no moral. All this is positively unmoral, except that aren't you ashamed to be doing any kind of dentistry and taking the bread right out of the specialists' mouths? I am, and would say more, except that I have got to stop and fill some root canals, which, when finished, I devoutly hope no one will X-ray.—*New Jersey Journal*, Dec., 1915.

